# Web-Based Personalized Nutrition System for Delivering Dietary Feedback Based on Behavior Change Techniques: Development and Pilot Study among Dietitians

**DOI:** 10.3390/nu13103391

**Published:** 2021-09-27

**Authors:** Kentaro Murakami, Nana Shinozaki, Shizuko Masayasu, M. Barbara E. Livingstone

**Affiliations:** 1 Department of Social and Preventive Epidemiology, School of Public Health, University of Tokyo, Tokyo 113-0033, Japan; nana-s@m.u-tokyo.ac.jp; 2 Ikurien-Naka, Ibaraki 311-0105, Japan; masayasu@ikurien.com; 3 Nutrition Innovation Centre for Food and Health (NICHE), School of Biomedical Sciences, Ulster University, Coleraine BT52 1SA, UK; mbe.livingstone@ulster.ac.uk

**Keywords:** personalized nutrition, behavior change, diet quality, dietary patterns, dietary feedback, dietary advice, acceptability, Meal-based Diet History Questionnaire, Internet, Japan

## Abstract

Given the complex and varied nature of individual characteristics influencing dietary behaviors, personalized dietary advice may be more effective than generalized “one-size-fits-all” advice. In this paper, we describe a web-based personalized nutrition system for improving the quality of overall diet in the general adult population. The development process included identification of appropriate behavior change techniques, modification of dietary assessment method (Meal-based Diet History Questionnaire; MDHQ), selection of dietary components, and a personalized dietary feedback tool. A pilot study was conducted online among 255 dietitians. Each completed the MDHQ, received his/her own dietary feedback report, and evaluated the relevance of the report based on 12 questions using a 5-point Likert scale from “totally disagree” (score 1) to “totally agree” (score 5). The mean value of overall acceptability score of dietary feedback report was 4.2. The acceptability score was, on average, higher in plausible energy reporters (compared with implausible energy reporters), participants who printed out the report (compared with those who did not), and those spending ≥20 min to read the report (compared with those spending <20 min). This is the first attempt to develop a web-based personalized nutrition system in Japan, where dietitians were broadly supportive of the dietary feedback report.

## 1. Introduction

Unhealthy diets pose a major risk for morbidity and premature death, and improving the quality of diet is now a global priority [1]. “One-size-fits-all” dietary guidelines do not achieve the changes in dietary behavior needed to achieve healthier dietary patterns [2]. Considering the complex and varied nature of individual characteristics that are related to dietary behaviors, targeted personalized dietary advice based on these characteristics may be more effective than generalized advice [3].

However, in Japan at present, no personalized dietary feedback system that integrates a dietary assessment tool with evidence-based behavior change techniques (BCTs) exists. Recent development of a novel dietary assessment tool based on detailed dietary behavior and intake data in the Japanese population [4] has motivated us to develop a web-based personalized nutrition (PN) system for improving the quality of overall diet in the general adult population. Combining the increasing utilization of the Internet as a method for delivering advice for behavioral changes, with web-based dietary assessment tools to provide targeted dietary advice at the individual level could provide a promising, cost-effective, and scalable approach for improving dietary behaviors [5,6,7].

In this paper, we describe the development process and piloting among dietitians of our web-based PN system for helping improve the quality of overall diet in the general adult population.

## 2. Materials and Methods

### 2.1. Development of Web-Based Personalized Nutrition System

**Figure 1:**
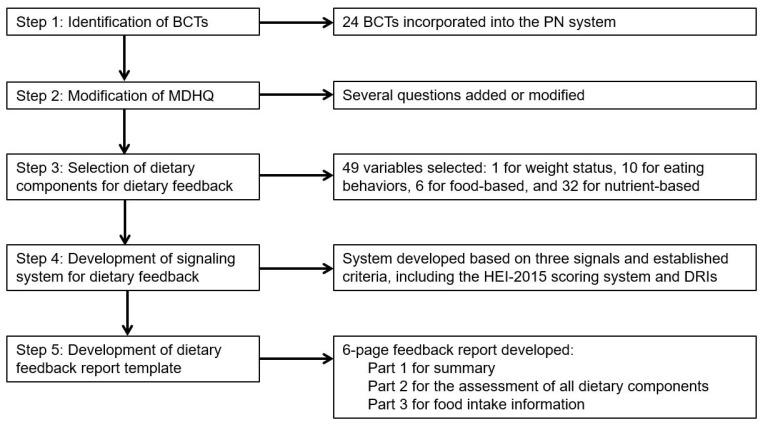
A summary of the development process of the personalized nutrition (PN) system. BCTs, behavior change techniques; DRIs, Dietary Reference Intakes; HEI-2015, Healthy Eating Index-2015; MDHQ, Meal-based Diet History Questionnaire.

Our web-based PN system was conceptualized with a view to improving the quality of overall diet in the general adult population. We incorporated validated BCTs into the PN system, as well as the framework of a future intervention trial, where the effectiveness of the dietary feedback can be assessed. The system consists of three major components: collection of dietary intake information using a recently developed Meal-based Diet History Questionnaire (MDHQ) [4]; dietary intake estimation and data conversion using a series of computer algorithms; and dietary feedback report accompanied by personalized dietary advice, based on well-established measures, including the Healthy Eating Index-2015 (HEI-2015) [8,9] and the Japanese Dietary Reference Intakes (DRIs) [10]. The development process of the PN system is summarized in Figure 1.

#### 2.1.1. Step 1: Identification of Behavior Change Techniques

Initially, we conducted a literature review to identify BCTs that would be fit-for-purpose in our PN system. For this, we selected the Food4Me BCT framework [11] as a starting point because of its compatibility with our concept (i.e., web-based and aiming at improving overall diet quality) [5,12,13,14,15,16]. The Food4Me BCT framework is based on well-established CALO-RE (Coventry, Aberdeen, and London-Refined) BCT taxonomy for physical activity and eating behaviors [17] and smoking cessation [18]. These taxonomies are underpinned by a variety of theoretical constructs of behavior change [19], including control theory [20], the information–motivation–behavioral skills model [21], the theory of planned behavior [22], and social cognitive theory [23]. Although a more recent, comprehensive, structured BCT taxonomy has been developed [24], we considered that the Food4Me BCT framework is more suitable for the domain of eating behaviors.

Of the 26 BCTs in the Food4Me framework, 3 BCTs (shaping; plan social support or social change; and use of follow-up prompts) were considered beyond the scope of our system and thus excluded. Conversely, a BCT not included in the Food4Me framework but included in CALO-RE taxonomy (provide formative information about others’ behavior) [17] was added. Consequently, a total of 24 BCTs were identified to incorporate into our PN system, as described in the Results section.

#### 2.1.2. Step 2: Modification of Dietary Assessment Method

In our PN system, dietary intake information is collected using the MDHQ. Details of the MDHQ have been published elsewhere [4]. Briefly, the MDHQ is a self-administered questionnaire designed for estimating food and nutrient intakes for each meal type separately (breakfast, morning snack, lunch, afternoon snack, dinner, and night snack). The MDHQ consists of the three different parts: questions on consumption frequency of 24 generic food groups for each meal type; questions on the relative consumption frequency of sub-food groups within one of the generic food groups; and questions on general eating behaviors. The reference time period is defined as the preceding month.

To improve the usefulness of information derived from the MDHQ in the PN system, we made the following modifications. First, questions on the number of each meal type consumed per week were added, because meal frequency was directly associated with overall diet quality in Japanese adults [25]. Second, the method for estimating alcoholic beverages was changed to a typical one based on consumption frequency and portion size (i.e., semiquantitative food frequency questionnaire style) to improve the clarity of questions and thus data quality. Third, we added questions on the amount of brown rice consumed and the consumption frequency of whole-grain bread, given the importance of whole grains for healthy eating [26]. Finally, we added questions on whether bread was consumed with jam, honey, etc. or with fat spread to improve the accuracy of estimation of added sugars and fat subtypes.

#### 2.1.3. Step 3: Selection of Dietary Components for Dietary Feedback

To achieve the ultimate purpose of our PN system, we considered that our dietary feedback should include a quantitative but easily understood measure of overall diet quality. For this, the HEI-2015 was selected. The HEI-2015 is a 100-point scale to assess compliance with the 2015–2020 Dietary Guidelines for Americans, with a higher score indicating a better quality of overall diet [8,9]. The efficacy of the HEI-2015 in assessing the overall diet quality of Japanese has been supported by our previous analyses [27,28]. Furthermore, it is well established that higher diet quality as assessed by established diet quality measures such as the HEI-2015 is associated with lower risk of all-cause mortality [29,30,31].

We also considered that our dietary feedback should include a wide range of dietary components. For this guide, we included components of the HEI-2015 (fruits, vegetables, dairy products, protein foods, whole grains, refined grains, sodium, added sugars, saturated fats, and the ratio of monounsaturated and polyunsaturated fats to saturated fats). We further included 28 nutrients, the reference values of which are published in the Japanese DRIs [10] and estimated intakes of which can be reliably obtained based on the Standard Tables of Food Composition in Japan [32]. Additionally, a series of eating pattern variables (e.g., meal frequency) and the HEI-2015 score for each meal were included, in addition to body mass index (BMI) as a measure of weight status.

Consequently, our PN system included the assessment of 1 weight status variable, 10 eating behavior variables (including HEI-2015 total score of total diet), 6 food-based variables, and 32 nutrient-based variables. Based on a series of ad hoc computer algorithms in the MDHQ, dietary intakes of 24 generic food groups and 88 food items (Table S1) were calculated. Intakes of energy and nutrients were calculated using food intake information and the Standard Tables of Food Composition in Japan [32]. Component scores needed for the calculation of HEI-2015 were calculated based on the Japanese version [27,33] of the US Food Patterns Equivalents Database [34]. To minimize the influence of dietary intake misreporting [35], all the dietary variables were energy-adjusted based on the density model. To make it possible to compare dietary intake values with the reference values derived from the Japanese DRIs [10], the energy-adjusted dietary variables were then standardized to the equivalent level of sex- and age-group specific estimated energy requirement for moderate physical activity [10] (mainly because of difficulty in obtaining an accurate measure of physical activity based on self-report). A comprehensive list of variables used in our PN system is available in Table S2.

#### 2.1.4. Step 4: Development of Signaling System for Dietary Feedback

The next step was to develop a signaling system for the assessment of each component. The signals used were red cross (meaning improvement strongly recommended), amber triangle (meaning improvement recommended), and blue circle (meaning good, and no change recommended), all of which are easy to understand intuitively for Japanese, without any explanation.

Cutoff values for the categorization, as shown in Table S2, were determined based on established criteria, whenever possible. Specifically, the categorization of weight status as assessed by BMI was based on the World Health Organization’s definition [36]. There are no established criteria for eating pattern variables, and thus, we subjectively determined cutoff values partly based on the distribution of each variable in Japanese adults [25,28]. For food-related variables (except for dairy products) and the ratio of monounsaturated and polyunsaturated fats to saturated fats, we determined cutoff values based on the scoring system of HEI-2015 [8]. For dairy products, the cutoff values on the HEI-2015 system were halved given the large difference in the reference values for calcium between Japan and the US (Estimated Average Requirement: 500–650 vs. 800–1100 mg/day; Recommended Dietary Allowance: 600–800 vs. 1000–1300 mg/day) [10,37].

Cutoff values for nutrients (except for added sugars and alcohol) were determined according to a predefined algorithm, using sex- and age group-specific reference values in the Japanese DRIs [10]. These were, depending on the availability of reference values, either a combination of Estimated Average Requirement and Recommended Dietary Allowance, recommended values for the prevention of chronic diseases (i.e., Tentative Dietary Goal for Preventing Lifestyle-related Diseases), or Adequate Intake (see Table S3). The cutoff values for added sugars and alcohol were determined by the World Health Organization’s definition [38] and a combined analysis of 83 prospective studies on the association between alcohol consumption and mortality [39], respectively.

#### 2.1.5. Step 5: Development of Dietary Feedback Report Template

Finally, we developed a 6-page dietary feedback report template that included all the contents described above, as well as personalized messages. The development of a total of 58 personalized messages was based on a variety of reputable sources [40,41,42,43,44,45,46,47,48]. Details of the feedback report are described in the Results section.

### 2.2. Pilot Study

#### 2.2.1. Overview and Study Population

A pilot study was conducted online among dietitians. The main purpose was to assess the acceptability of the dietary feedback report. The secondary purpose was to assess the comparability of the MDHQ with a well-validated dietary assessment questionnaire, namely the Brief-type Diet History Questionnaire (BDHQ) [33,49,50].

We invited dietitian members of the Japan Human Health and Nutrition Association to participate in this study by sending a newsletter followed by an invitation email (including URL for the survey site). The key inclusion criterion for this study of community dwelling (free-living) dietitians was their willingness to complete all the measurements. Excluded from the study were those who had experienced dietary counseling from a doctor or dietitian, those taking insulin treatment for diabetes, those taking dialysis treatment, those without sufficient Internet access, pregnant or lactating women, and those aged <20 years, as well as non-dietitians. Of 318 dietitians invited, 255 (80%) competed the survey.

The study was conducted according to the guidelines of the Declaration of Helsinki and approved by the Ethics Committee of the University of Tokyo, Faculty of Medicine (protocol code: 2021041NI; date of approval: 27 May 2021). Informed consent was obtained online from all subjects involved in the study.

#### 2.2.2. Survey Procedure

The survey was conducted in July and August 2021, and all data were collected online using Google Forms. First, the participants answered the MDHQ. Information on date of birth (to calculate age) and self-reported body height and weight was also collected in the MDHQ. Usually immediately after the completion of the MDHQ, the participants answered the BDHQ; the order of administration of the MDHQ and BDHQ was fixed. This is a validated questionnaire for assessing dietary habits during the preceding month and has been used in numerous epidemiologic studies in Japan [51,52,53]. Detailed descriptions on the BDHQ as well as its validity are available elsewhere [33,49,50].

Within a week of completion of the dietary questionnaires, we sent each participant his/her feedback report (PDF file) as an email attachment. In the email, only a brief description of each individual’s result was provided (i.e., HEI-2015 total score followed by the personalized presentation of three most relevant components for the improvement of overall diet quality and a piece of personalized advice on the amount of foods consumed based on weight status). After reviewing his/her own feedback report, each participant was asked to visit a survey site (URL provided in the email) and answer a series of questions regarding acceptability of the dietary feedback report (i.e., acceptability questionnaire). All the participants completed the acceptability questionnaire within three weeks after receiving the dietary feedback report.

#### 2.2.3. Assessment of Acceptability of the Dietary Feedback Report

The acceptability of the dietary feedback report was assessed using 12 questions, which were derived from existing questionnaires [54,55], with slight modifications. Answers were rated on a 5-point Likert scale from “totally disagree” to “totally agree”. Each component was scored using the scores of 1 to 5, with higher scores indicating higher acceptability. The participants also could provide reason(s) for each of their answers (in open-end formats); this information was not examined in the present paper because it will be utilized separately in future research. The overall acceptability score was calculated as the average of 12 components. We also asked about the device used for reading the dietary feedback report (e.g., paper, PC, tablet, smart phone, etc.) and time spent reading the feedback report.

#### 2.2.4. Statistical Analysis

All the analyses were conducted using SAS statistical software (version 9.4, SAS Institute Inc., Cary, NC, USA). Data are shown using means and standard deviations for continuous variables and numbers and percentages of participants for categorical variables. The comparability of MDHQ with BDHQ was assessed concerning energy intake and overall diet quality (assessed as the HEI-2015). To assess the estimation ability at the group level, mean values derived from the MDHQ were compared with those derived from the BDHQ on the basis of each paired *t*-test. Pearson correlation coefficients were used to assess the ability to rank the individuals in a population. In addition, agreement between the MDHQ and BDHQ was assessed on the basis of the Bland–Altman analysis [56].

Acceptability of the dietary feedback report was examined in relation to several participant characteristics as well as in the overall population. Participant characteristics considered were selected a priori and included age (divided by median), weight status, overall diet quality (divided by median), dietary reporting status, whether the dietary feedback report was printed (no or yes), and time spent reading the report (<20 or ≥20 min; 20 min considered the minimum time for thorough reading based on pilot testing). Weight status was categorized on the basis of BMI: underweight (<18.5 kg/m^2^), normal weight (≥18.5 to <25 kg/m^2^), or overweight (≥25 kg/m^2^) [36]. Dietary reporting status was categorized on the basis of reported energy intake to sex- and age-specific estimated energy requirement [10]: implausible (<0.7 or >1.3) or plausible (0.7 to 1.3). Differences in acceptability score of the dietary feedback report between each category of respective variables were examined using independent *t*-test, except for weight status for which analysis of variance was followed by Bonferroni’s post hoc test. We considered *p* values < 0.05 statistically significant.

## 3. Results

### 3.1. Dietary Feedback Report

**Figure 2:**
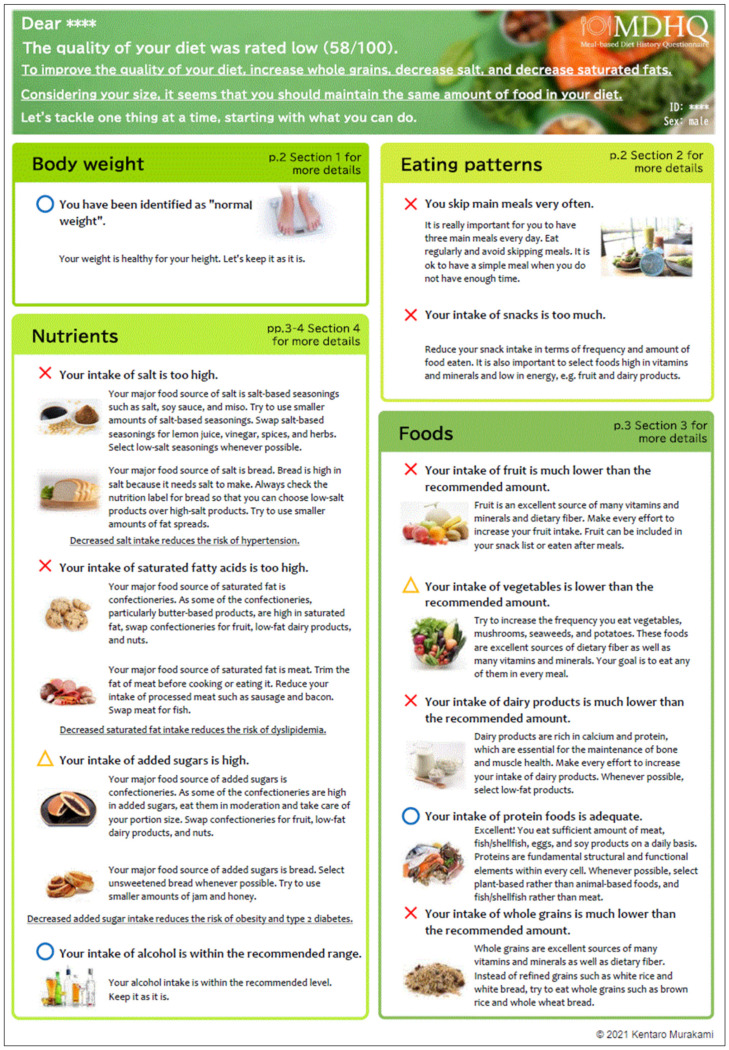
Part 1 (page 1) of the dietary feedback report. This is a summary of feedback accompanied by personalized messages. On the top box, an assessment of overall diet quality, as assessed by the Healthy Eating Index-2015 total score for the total diet, is shown, as well as the Big 3, i.e., three most relevant components for the improvement of overall diet quality, and a piece of advice on the amount of foods consumed based on weight status. Shown below the top box are qualitative assessment and personalized messages for 12 selected components, divided by categories of weight status, eating patterns, nutrient intake, and food group intake. Here, the translated version is shown; the original is in Japanese.

The six-page dietary feedback report consisted of three parts: summary of feedback report accompanied by personalized messages (Part 1; an example shown in Figure 2), quantitative assessment of all dietary components (Part 2; an example shown in Figure S1), and quantitative information on food intake for each eating occasion (Part 3; an example shown in Figure S2). Whenever possible, we tried to incorporate relevant BCTs into the feedback report contents (Table S4). Briefly, a total of 15 BCTs were incorporated into Part 1 of the dietary feedback report: “provide information on consequences of behavior to the individual” (code 2); “provide information about others’ approval” (code 3); “provide rewards contingent on successful behavior” (code 4); “provide feedback on performance” (code 5); “goal-setting (behavior)” (code 6); “set graded tasks” (code 8); “prompt review of behavioral goals” (code 9); “prompt review of outcome goals” (code 10); “action planning “ (code 12); “barrier identification or problem solving” (code 13); “prompt practice” (code 14); “emphasize choice” (code 15); “tailor interactions appropriately” (code 16); “provide instruction on how to perform the behavior” (code 21); and “provide information on where and when to perform the behavior” (code 23). Incorporated into Part 2 of the dietary feedback report were: “provide information on consequences of behavior in general” (code 1); “goal setting (outcome)” (code 7); “prompt self-monitoring of behavioral outcome” (code 11); and “provide formative information about others’ behavior” (code 24), as well as codes 9, 10, and 21. Incorporated into Part 3 of the dietary feedback report were codes 5 and 16.

#### 3.1.1. Part 1 of Dietary Feedback Report

The first page (Figure 2) was the summary of dietary feedback report, including qualitative assessment of 12 selected components (by showing either red cross, amber triangle, or blue circle). We selected BMI because of its importance to overall health status. Meal frequency and snack energy were selected based on previous observations in Japanese adults: the former’s direct association with overall diet quality [25] and the latter’s dependence on energy-dense and nutrient-poor foods such as confectioneries and sugar-sweetened beverages [28]. We selected the four nutrients (sodium, saturated fats, added sugars, and alcohol), excessive intakes of which are of concern, because we considered that it is more important and meaningful to show personalized messages determined by the major food contributors of such nutrients in order to reduce intakes (while personalized messages on major food contributors may not necessarily be useful in order to increase intakes because food sources do not matter in such cases). On the other hand, we selected the five foods (fruits, vegetables, dairy products, protein foods, and whole grains), insufficient intakes of which are of concern. In this way, some improvement at the food level, we assumed, would also make improvement for other nutrients not shown in this page, insufficient intakes of which are of concern.

For each component, the personalized messages consisted of the main assessment message and more specific sub message(s). With regard to weight status, five main/sub messages were prepared, with an example:

- “You have been identified as overweight.” (main assessment message)
- “You will become healthier if you succeed in a slight decrease in your body weight. Gradually decrease the amount of food you eat and become more physically active.” (more specific sub messages)

Similarly, for each of eating pattern and food variables as well as alcohol, three main/sub messages were prepared. An example of meal frequency was as follows:

- “You skip main meals very often.” (main assessment message)
- “It is really important for you to have three main meals every day. Eat regularly and avoid skipping meals. It is ok to have a simple meal when you do not have enough time.” (more specific sub messages)

Three main messages were similarly prepared for sodium, saturated fats, and added sugars (e.g., “Your intake of added sugars is too high.”), while a different algorithm was used for sub messages. Depending on the two top food contributors, two messages (out of eight for sodium, six for saturated fats, and six for added sugars) accompanied the main message. These were prepared as practical food-based tips to improve nutrient intakes, with an example of “Your major food source of salt is salt-based seasonings such as salt, soy sauce, and miso. Try to use smaller amounts of salt-based seasonings. Swap salt-based seasonings for lemon juice, vinegar, spices, and herbs. Select low-salt seasonings whenever possible.”

In addition, on the top of this page, the assessment of HEI-2015 score of total diet (as a measure of overall diet quality) was shown. This was followed by the personalized presentation of “Big 3” (three most relevant components for the improvement of overall diet quality) and a piece of personalized advice on the amount of foods consumed based on weight status. Potential candidates (and priority orders) for the Big 3 were whole grains, sodium, saturated fats, fruit, vegetables, added sugars, protein foods, and dairy products, which was determined based on the current concern of the Japanese diet [27].

#### 3.1.2. Part 2 of Dietary Feedback Report

The second to fourth pages of the dietary feedback report (Figure S1) included quantitative (as well as qualitative) assessment of all dietary components. The results on dietary intake were shown in comparison with the cutoff values determined, which were accompanied by the signal (i.e., red cross, amber triangle, and blue circle). All the variables assessed here as well as the assessment criteria are shown in Table S2. For each of the dietary components, a general comment was also provided (e.g., “A higher intake of vegetables has been shown to be associated with a lower risk of cardiovascular diseases.”). Furthermore, some examples of one typical portion were shown for each food component, while a list of major food sources was provided for each nutrient component. Thus, this part only provided feedback but not any personalized messages.

#### 3.1.3. Part 3 of Dietary Feedback Report

The fifth and sixth pages of the dietary feedback report (Figure S2) included quantitative information on food intake assessed by the MDHQ (24 generic food groups and 88 food items; see Table S1) for each eating occasion (i.e., breakfast, morning snack, lunch, afternoon snack, dinner, and night snack), as well as all the answers in the MDHQ. This was designed to inform the participant how and when he/she ate each food so that he/she can get tips to modify their dietary behaviors. Again, this part only provided feedback but not any personalized messages.

### 3.2. Pilot Study

The pilot study included 255 dietitians (six males and 249 females) aged 23–70 years (Table 1). The mean age was 49 years and the mean BMI was 22.1 kg/m^2^. The prevalence of implausible energy reporters was 26%. About half of participants (48%) printed out their own dietary feedback reports, with the remaining participants accessing the report on an electronic device, while 37% of participants spent ≥20 min for reading the report.

#### 3.2.1. Comparability of the MDHQ with the BDHQ

Table 2 shows the energy intake and the HEI-2015 scores derived from the MDHQ and BDHQ. There was no significant difference in mean energy intake and HEI-2015 total score between the two questionnaires. The Pearson correlation coefficient was 0.56 for energy and 0.46 for HEI-2015 total score (both *p* < 0.0001). For HEI-2015 components, compared with the BDHQ, the MDHQ provided higher mean scores of whole grains and sodium and lower mean scores of greens and beans, total protein foods, seafood and plant proteins, added sugars, and saturated fats. The Pearson correlation coefficients ranged from −0.04 (whole grains) to 0.71 (whole fruits) with a median of 0.38.

**Figure 3:**
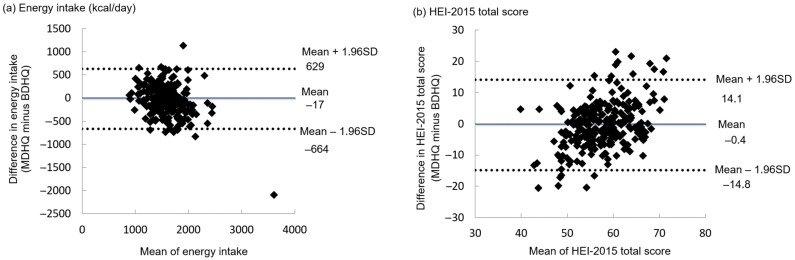
Bland–Altman plots assessing the agreement between the Meal-based Diet History Questionnaire (MDHQ) and Brief-type Diet History Questionnaire (BDHQ) in terms of (**a**) energy intake and (**b**) the Healthy Eating Index-2015 (HEI-2015) total score (*n* = 255). SD, standard deviation.

Bland–Altman plots were applied for assessing the agreement between the MDHQ and BDHQ in terms of energy intake and the HEI-2015 total score (Figure 3). The mean difference was small, suggesting good agreement at the group level. However, the limit of agreement (mean difference ± 1.96 standard deviation) was wide, indicating poor agreement at the individual level. For the HEI-2015 component scores (Table 2), the mean difference was generally small (except for whole grains and fatty acids), suggesting good agreement at the group level. However, the limit of agreement varied depending on variables. For example, good agreement at the individual level was found for total protein foods and seafood and plant proteins. Conversely, relatively poor agreement at the individual level was observed for whole grains and refined grains. The exclusion of implausible energy reporters of MDHQ did not change the results materially (data not shown), except for higher energy intakes in both MDHQ and BDHQ (mean: 1678 and 1676 kcal/day, respectively).

#### 3.2.2. Acceptability of the Dietary Feedback Report

Acceptability scores of the dietary feedback report are shown in Table 3. Overall, the acceptability was encouraging, with 10 out of 12 components as well as the total score having a mean score of 4 or more (the maximum score 5). The remaining components were the length and correctness of the report (mean score: 3.3 and 3.9, respectively).

As shown in Table S5, the acceptability of the dietary feedback report was, on average, higher in plausible energy reporters (compared with implausible energy reporters), participants who printed out the report (compared with those who did not), and those spending ≥20 min reading the report (compared with those spending <20 min). In contrast, age, weight status, and overall diet quality were not clearly associated with the acceptability of the dietary feedback report (Table S6).

## 4. Discussion

To our knowledge, this is the first attempt to develop a web-based PN system using BCTs to deliver dietary feedback aimed at improving overall diet quality in Japan. While there are a few studies where personalized dietary feedback has been developed (and evaluated) [5,6,7], with the exception of the Food4Me dietary feedback system [5], the development process has rarely been described in sufficient detail to permit an informed evaluation of their likely efficacy. In the Food4Me study, dietary information was collected using an online food frequency questionnaire, which was validated against a 4-day dietary record [16]. The dietary feedback system was developed based on well-established criteria for various dietary variables, including the Healthy Eating Index (2010 version) [5]. Additionally, a total of 26 BCTs were incorporated into the system, detailed descriptions of which have been available [11]. Our development process was largely consistent with the Food4Me system.

Accurate assessment of dietary intake at the individual level is prerequisite for delivering personalized dietary feedback and advice. In our pilot study, there was no significant difference in the HEI-2015 total score between the MDHQ and a well-validated dietary assessment questionnaire (BDHQ). Furthermore, the Pearson correlation coefficient of HEI-2015 total score between the MDHQ and BDHQ was 0.46 (*p* < 0.0001), suggesting a moderate correlation. Our results are consistent with previous studies, where a novel dietary assessment questionnaire was compared with an established dietary assessment questionnaire for measuring overall diet quality [57,58,59,60,61]. For example, our previous study showed that the Spearman correlation coefficient of the HEI-2015 total score between a food combination questionnaire and the BDHQ was 0.49 [57]. The correlation coefficients observed in other studies ranged from 0.38 to 0.61 [58,59,60,61].

However, the Bland–Altman plot showed poor agreement between the MDHQ and BDHQ in terms of the HEI-2015 total score at the individual level, despite the negligible mean difference. This might not necessarily mean that estimates derived from the MDHQ are unreliable, given poor agreement between the BDHQ and 16-day weighed dietary record at least at the individual level (limit of agreement: −7.9, 13.7 for women and −9.8, 14.3 for men) [33]. For a number of reasons, we consider that the MDHQ is in principle superior to the BDHQ. First, the MDHQ is data-driven, so the development of the questionnaire structure, food items, and dietary intake calculation algorithms was based on detailed dietary information derived from a 16-day weighed dietary record obtained from 242 Japanese adults [4]. Second, because the MDHQ assesses dietary intake on each eating occasion separately, information on various aspects of dietary behaviors is collected (e.g., meal frequency and percentage of energy from snacking occasions), which is invaluable for personalized dietary feedback and advice. Third, given that the cognitive tasks required during dietary recall are complex, including understanding what information is being asked for, and searching for and evaluating the retrieved information before providing a response [62], the MDHQ may be easier to answer, facilitating better estimation of food intake. This may be particularly relevant to Japanese because previous studies among Japanese adults have shown that the selection, amount, and combination of foods consumed are markedly different between meal types [57,63,64,65]. The present study suggests that the MDHQ may have sufficient ability to evaluate the overall diet quality at least at the group level, in comparison with the BDHQ. A rigorous evaluation of the validity of the MDHQ against a 4-day weighed dietary record is now being conducted among the general adult population to confirm this observation.

In our pilot study, the acceptability of the dietary feedback report was, on average, sufficient (mean > 4 based on the 5-point Likert scale) for 10 of the 12 components. For many participants, the report was considered believable, relevant, interesting, logical, understandable, well formulated, complete, and personal. In addition, many participants considered that “I will use the report” and “the report will help me to eat healthier”. In contrast, some concerns were raised regarding the length and (perceived) correctness of the report. The concern about the length of the report was not clearly related to any participant characteristics examined. In our dietary feedback report, the most relevant and personalized information was summarized on the first page (Part 1), with the remaining five pages (Parts 2 and 3) having detailed but supplemental information. Thus, it may be better in the future that each part is prepared in separate files so that each participant can self-determine if he/she will have access to the second and third parts of dietary feedback report. For the correctness of the report, the acceptability score was lower among participants whose reported energy intake was considered implausible, who did not print out the feedback report, and who spent <20 min to read the feedback report. It may be possible that some participants could have been on a weight-reducing diet so their reported intake was more accurate than assessed, which may have led to the perceived inaccuracies in the dietary feedback report; unfortunately, we did not collect information on whether consuming a weight reducing diet in this study. In any case, throughout the pilot study, participants were not provided with any information or recommendation related to these aspects. For future use, it may be important to try to enhance participants’ buy-in not only before and during the dietary assessment but also at the stage of delivering dietary feedback report.

However, findings on the acceptability of dietary feedback report should be interpreted cautiously, mainly because the pilot study was conducted among a group of dietitians rather than the general populations. Our sample is also unlikely to represent the whole dietitian population given the large number of new dietitians licensed (*n* > 25,000 in each of the recent 15 years; information on the total number of dietitians is not available) [66]. Considering that health consciousness and nutrition literacy, as well as buy-in, of dietitians are likely to be high, somewhat different results might be obtained in general populations (or the representative dietitian population). Nevertheless, we consider that sufficient acceptability by experts is an essential first step for our dietary feedback report.

Although our PN system was developed for Japanese, the development was based on actual food consumption data, and the process was highly standardized; thus, the same strategy could also be applied for other populations. In addition, while the MDHQ is designed to collect dietary information for each eating occasion, this detailed information was rarely used in the dietary feedback report mainly because the validity in this regard has not been established. An ongoing validation study of the MDHQ against the dietary record will establish, or otherwise, the utility of the MDHQ at the eating occasion level and ultimately facilitate more personalized feedback to participants.

## 5. Conclusions

Here, we described a systematic process of the development of our web-based PN system for helping improve the quality of overall diet in the general adult population. The development process included the identification of BCTs, modification of dietary assessment method (MDHQ), selection of dietary components, development of signaling system for dietary feedback, and development of dietary feedback report. This detailed information is valuable for future research with similar topics. In our pilot study, dietitians were broadly supportive of such a dietary feedback report. These results warrant a more comprehensive evaluation of the effectiveness of this PN system while also conducting a validation study of the MDHQ against weighed dietary record.

## Figures and Tables

**Table 1:** Basic characteristics of study population (*n* = 255) ^1^.

Variable	Value
Sex, *n* (%)	
Male	6 (2.4)
Female	249 (97.7)
Age (years)	48.6 ± 10.8
Body height (cm) ^2^	158.6 ± 5.8
Body weight (kg) ^2^	55.5 ± 8.5
BMI (kg/m^2^) ^3^	22.1 ± 3.2
Weight status, *n* (%)	
Underweight (BMI < 18.5 kg/m^2^)	27 (10.6)
Normal weight (BMI ≥ 18.5 to <25 kg/m^2^)	183 (71.8)
Overweight (BMI ≥25 kg/m^2^)	45 (17.7)
Dietary reporting status in the MDHQ, *n* (%)	
Implausible ^4^	65 (25.5)
Plausible ^5^	190 (74.5)
Whether the MDHQ feedback report was printed, *n* (%)	
No	133 (52.2)
Yes	122 (47.8)
Time spent reading the MDHQ feedback report, *n* (%)	
Less than 20 min	162 (63.5)
20 min or more	93 (36.5)

BMI, body mass index; MDHQ, Meal-based Diet History Questionnaire. ^1^ Values are means ± standard deviations unless otherwise indicated. ^2^ Based on self-report. ^3^ Calculated using self-reported body weight and height. ^4^ Including 63 under-reporters and two over-reporters. Under- and over-reporters were defined as participants with the ratio of reported energy intake to estimated energy requirement of <0.7 and >1.3, respectively. ^5^ Defined as participants with the ratio of reported energy intake to estimated energy requirement of 0.7 to 1.3.

**Table 2:** Energy intake and the HEI-2015 scores calculated based on the MDHQ and BDHQ (*n* = 255) ^1^.

Variable	Maximum Score	MDHQ	BDHQ	Pearson Correlation ^2^	Mean Difference ^3^	Limit of Agreement ^4^
Energy intake (kcal/day)	---	1581 ± 283	1598 ± 392	0.56 ****	−17	629, −664
HEI-2015 total score	100	57.2 ± 8.1	57.5 ± 5.5	0.46 ****	−0.4	14.1, −14.8
HEI-2015 component score	---	---	---	---	---	---
Total fruits	5	2.2 ± 1.3	2.2 ± 1.3	0.65 ****	0.0	2.1, −2.1
Whole fruits	5	3.4 ± 1.6	3.3 ± 1.6	0.71 ****	0.1	2.5, −2.3
Total vegetables	5	4.8 ± 0.7	4.9 ± 0.4	0.31 ****	−0.1	1.3, −1.5
Greens and beans	5	3.7 ± 1.5	4.3 ± 1.2 ***	0.39 ****	−0.7	2.3, −3.6
Whole grains	10	2.8 ± 2.6	0.4 ± 0.5 ***	−0.04	2.4	7.5, −2.8
Dairy	10	2.6 ± 1.5	2.5 ± 1.6	0.62 ****	0.1	2.7, −2.5
Total protein foods	5	4.5 ± 0.6	5.0 ± 0.2 ***	0.21 ***	−0.5	0.7, −1.6
Seafood and plant proteins	5	4.9 ± 0.5	5.0 ± 0.2 **	0.46 ****	−0.1	0.7, −0.9
Fatty acids	10	6.9 ± 2.4	8.3 ± 1.9 ***	0.32 ****	−1.4	3.6, −6.4
Refined grains	10	1.6 ± 2.3	1.5 ± 2.1	0.32 ****	0.1	5.2, −4.9
Sodium	10	1.5 ± 2.2	1.0 ± 1.8 **	0.43 ****	0.5	4.8, −3.8
Added sugars	10	9.3 ± 1.1	9.8 ± 0.6 ***	0.38 ****	−0.5	1.5, −2.5
Saturated fats	10	8.9 ± 1.4	9.4 ± 1.0 ***	0.23 ***	−0.5	2.5, −3.5

BDHQ, Brief-type Diet History Questionnaire; HEI-2015, Healthy Eating Index-2015; MDHQ, Meal-based Diet History Questionnaire. ^1^ Values are means ± standard deviations unless otherwise indicated. Mean values derived from the MDHQ were compared with those derived from the BDHQ based on paired *t*-test: ** *p* < 0.01; *** *p* < 0.001. ^2^ Pearson correlation coefficients between the MDHQ and BDHQ values: *** *p* < 0.001; **** *p* < 0.0001. ^3^ Calculated as mean of difference in the MDHQ value minus the BDHQ value. ^4^ Calculated as the mean difference ± 1.96 standard deviation.

**Table 3:** Acceptability scores of the MDHQ feedback report (*n* = 255) ^1^.

Variable	Value
Total score	4.2 ± 0.4
The report is believable	4.1 ± 0.6
The report is relevant	4.3 ± 0.6
The report is interesting	4.6 ± 0.6
The report is logical	4.3 ± 0.7
The report is understandable	4.3 ± 0.7
The report is well formulated	4.4 ± 0.7
The report is complete	4.5 ± 0.6
The report is too long	3.3 ± 1.1
The report is personal	4.4 ± 0.7
The report is correct	3.9 ± 0.7
I will use the report	4.3 ± 0.7
The report will help me to eat healthier	4.5 ± 0.6

MDHQ, Meal-based Diet History Questionnaire. ^1^ Values are means ± standard deviations. Acceptability scores range from 1 (“strongly disagree”) to 5 (“strongly agree”), except for the item “The report is too long” for which the score is assigned vice versa. The total score was calculated as the average of all 12 items.

## Data Availability

The data presented in this study are available on request from the corresponding author. The data are not publicly available due to privacy or ethical restrictions.

## Supplementary Materials

The following are available online at https://www.mdpi.com/article/10.3390/nu13103391/s1, Table S1: List of food groups and food items estimated from the Meal-based Diet History Questionnaire, Table S2: Dietary variables and their assessment criteria shown in the second, third, and fourth pages of the dietary feedback report, Table S3: Dietary reference values used in the dietary feedback report, Figure S1: Part 2 (pages 2–4) of the dietary feedback report, Figure S2: Part 3 (pages 5–6) of the dietary feedback report, Table S4: Behavior change techniques (BCTs) used in the personalized nutrition system, Table S5: Acceptability scores of the MDHQ feedback report according to dietary reporting status, whether the feedback report was printed, and time spent reading the feedback report, Table S6: Acceptability scores of the MDHQ feedback report according to age, weight status, and diet quality.
