# Supplementary material for: Web-Based Personalized Nutrition System for Delivering Dietary Feedback Based on Behavior Change Techniques: Development and Pilot Study among Dietitians
Source: Nutrients. 2021 Sep 27;13(10):3391. doi: 10.3390/nu13103391 (PMC8538565; doi:10.3390/nu13103391)
Supplement: Supplementary file 1 [file nutrients-13-03391-s001.zip › nutrients-1389891-supplementary.pdf]

Table S1. List of food groups and food items estimated from the Meal-based Diet History Questionnaire.

Food group	Food item
Rice	White rice; brown rice
Bread	NA
Noodles	Wheat noodles; Chinese noodles; instant noodles; spaghetti; buckwheat noodles
Breakfast cereals	NA
Miso soup	NA
Pickled vegetables	NA
Meat	Liver; processed meat; beef; chicken; pork
Fish and shellfish	Small fish with bones; dried fish; canned tuna; salmon; white meat fish; oily fish; red meat fish; squid and octopus; eel; shrimp and crab; shellfish; fish eggs; ground fish meat products
Eggs	NA
Dairy products	Low-fat milk; full-fat milk; yogurt; cheese; ice cream
Pulses and nuts	Tofu (i.e., soybean curd); natto (i,e., fermented soybeans); tofu products; soy milk; peanuts and nuts
Vegetables	Cabbage; cucumbers; lettuce; bitter melon; burdock; radishes; onions; carrots; pumpkins; tomatoes; eggplants; green peppers; broccoli; Chinese cabbage; green leafy vegetables; bean sprouts; edamame (i.e., immature soybeans) and peas; mushrooms; seaweeds
Potatoes	NA
Fruits	Strawberries; persimmons; citrus; kiwi fruit; water melons; pears; bananas; grapes; melons; peaches; apples
Confectioneries	Rice crackers; Japanese sweets; candies, caramels, and chewing gum; Japanese bread with a sweet filling; snacks made from wheat flour; jellies; chocolates; biscuits and cookies; cakes
Water	NA
Green tea	NA
Barley tea	NA
Oolong tea	NA
Black tea	NA
Coffee	NA
Soft drinks	NA
Fruit and vegetable juice	NA
Alcoholic beverages	Beer; sake; shochu (i.e., Japanese distilled beverages); wine; whiskey and other spirits
Seasonings	Sugar added to coffee and tea; salt for cooking; vegetable oils; sugar for cooking; salt for soup; soy sauce; jam for bread; fat spread for bread; mayonnaise and dressing

NA, not applicable.

Table S2. Dietary variables and their assessment criteria used in the personalized nutrition system.

	Category 1 (red cross)		Category 2 (amber triangle)		Category 3 (blue circle)		Category 4 (amber triangle)		Category 5 (red cross)	
**Category and component**	**Cutoff point**	**Assessment**	**Cutoff point**	**Assessment**	**Cutoff point**	**Assessment**	**Cutoff point**	**Assessment**	**Cutoff point**	**Assessment**
Weight status [1]										
Body mass index (kg/m^2)	<17	Moderate and severe thinness	≥17 to <18.5	Underweight	≥18.5 to <25	Normal weight	≥25 to <30	Overweight	≥30	Obese
Eating pattern [2]										
Meal frequency (times/d)	<2.43 (= 17/7)	Very low	≥2.43 to <3	Low	3	Recommended				
Breakfast energy (% of total energy)	<10	Very low	≥10 to <20	Low	≥20	Recommended				
Lunch energy (% of total energy)	<15	Very low	≥15 to <25	Low	≥25 to ≤40	Recommended	>40 to <50	High	≥50	Very high
Dinner energy (% of total energy)	<20	Very low	≥20 to <30	Low	≥30 to ≤45	Recommended	>45 to <55	High	≥55	Very high
Snack energy (% of total energy)					≤10	Recommended	>10 to <20	High	≥20	Very high
Breakfast quality (HEI-2015 score)	<50	Very low	≥50 to <70	Low	≥70	Recommended				
Lunch quality (HEI-2015 score)	<50	Very low	≥50 to <70	Low	≥70	Recommended				
Dinner quality (HEI-2015 score)	<50	Very low	≥50 to <70	Low	≥70	Recommended				
Snack quality (HEI-2015 score)	<50	Very low	≥50 to <70	Low	≥70	Recommended				
Total diet quality (HEI-2015 score)	<50	Very low	≥50 to <70	Low	≥70	Recommended				
Food [3]										
Total fruits (cup Eq/1000 kcal)	<0.4	Very low	≥0.4 to <0.72	Low	≥0.72	Recommended				
Total vegetables (cup Eq/1000 kcal)	<0.55	Very low	≥0.55 to <0.99	Low	≥0.99	Recommended				
Dairy products (cup Eq/1000 kcal)	<0.33	Very low	≥0.33 to <0.59	Low	≥0.59	Recommended				
Total protein foods (ounce Eq/1000 kcal)	<1.25	Very low	≥1.25 to <2.25	Low	≥2.25	Recommended				
Whole grains (ounce Eq/1000 kcal)	<0.75	Very low	≥0.75 to <1.35	Low	≥1.35	Recommended				
Refined grains (ounce Eq/1000 kcal)					≤2.05	Recommended	>2.05 to ≤3.05	High	>3.05	Very high
Nutrient [4]										
Protein (g/d)	<EAR	Very low	≥EAR to <RDA	Low	≥RDA	Recommended				
Protein (% of total energy)	<0.7 × LLDG	Very low	≥0.7 × LLDG to <LLDG	Low	LLDG to ULDG	Recommended	>ULDG to <1.3 × ULDG	High	≥1.3 × ULDG	Very high
Fat (% of total energy)	<0.7 × LLDG	Very low	≥0.7 × LLDG to <LLDG	Low	LLDG to ULDG	Recommended	>ULDG to <1.3 × ULDG	High	≥1.3 × ULDG	Very high
SFA (% of total energy)	<0.7 × LLDG	Very low	≥0.7 × LLDG to <LLDG	Low	LLDG to ULDG	Recommended	>ULDG to <1.3 × ULDG	High	≥1.3 × ULDG	Very high

n-3 PUFA (g/d)	<0.7 × AI	Very low	≥0.7 × AI to <0.9 × AI	Low	≥0.9 × AI	Recommended				
n-6 PUFA (g/d)	<0.7 × AI	Very low	≥0.7 × AI to <0.9 × AI	Low	≥0.9 × AI	Recommended				
Ratio of PUFA + MUFA to SFA[3]	<1.25	Very low	≥1.25 to <2.25	Low	≥2.25	Recommended				
Carbohydrate (% of total energy)	<0.7 × LLDG	Very low	≥0.7 × LLDG to <LLDG	Low	LLDG to ULDG	Recommended	>ULDG to <1.3 × ULDG	High	≥1.3 × ULDG	Very high
Added sugars (% of total energy) [5]					<5	Recommended	≥5 to <10	High	≥10	Very high
Alcohol (g/week) [6]					≤100	Recommended	>100 to ≤200	High	>200	Very high
Dietary fiber (g/d)	<0.7 × LLDG	Very low	≥0.7 × LLDG to <LLDG	Low	≥LLDG	Recommended				
Sodium (NaCI g/d)					<ULDG	Recommended	≥ULDG to <1.3 × ULDG	High	≥1.3 × ULDG	Very high
Potassium (mg/d)	<0.7 × LLDG	Very low	≥0.7 × LLDG to <LLDG	Low	≥LLDG	Recommended				
Calcium (mg/d)	<EAR	Very low	≥EAR to <RDA	Low	≥RDA	Recommended				
Magnesium (mg/d)	<EAR	Very low	≥EAR to <RDA	Low	≥RDA	Recommended				
Phosphorus (mg/d)	<0.7 × AI	Very low	≥0.7 × AI to <0.9 × AI	Low	≥0.9 × AI	Recommended				
Iron (mg/d)	<EAR	Very low	≥EAR to <RDA	Low	≥RDA	Recommended				
Zinc (mg/d)	<EAR	Very low	≥EAR to <RDA	Low	≥RDA	Recommended				
Copper (mg/d)	<EAR	Very low	≥EAR to <RDA	Low	≥RDA	Recommended				
Manganese (mg/d)	<0.7 × AI	Very low	≥0.7 × AI to <0.9 × AI	Low	≥0.9 × AI	Recommended				
Vitamin A (µg/d)	<EAR	Very low	≥EAR to <RDA	Low	≥RDA	Recommended				
Vitamin D (µg/d)	<0.7 × AI	Very low	≥0.7 × AI to <0.9 × AI	Low	≥0.9 × AI	Recommended				
Vitamin E (mg/d)	<0.7 × AI	Very low	≥0.7 × AI to <0.9 × AI	Low	≥0.9 × AI	Recommended				
Vitamin K (µg/d)	<0.7 × AI	Very low	≥0.7 × AI to <0.9 × AI	Low	≥0.9 × AI	Recommended				
Thiamin (mg/d)	<EAR	Very low	≥EAR to <RDA	Low	≥RDA	Recommended				
Riboflavin (mg/d)	<EAR	Very low	≥EAR to <RDA	Low	≥RDA	Recommended				
Niacin (mg/d)	<EAR	Very low	≥EAR to <RDA	Low	≥RDA	Recommended				

Vitamin B-6 (mg/d)	<EAR	Very low	≥EAR to <RDA	Low	≥RDA	Recommended
Vitamin B-12 (μg/d)	<EAR	Very low	≥EAR to <RDA	Low	≥RDA	Recommended
Folate (μg/d)	<EAR	Very low	≥EAR to <RDA	Low	≥RDA	Recommended
Pantothenic acid (mg/d)	<0.7 × AI	Very low	≥0.7 × AI to <0.9 × AI	Low	≥0.9 × AI	Recommended
Vitamin C (mg/d)	<EAR	Very low	≥EAR to <RDA	Low	≥RDA	Recommended

AI, Adequate Intake; d, day; EAR, Estimated Average Requirement; Eq, equivalents; HEI-2015, Healthy Eating Index-2015; LLDG, lower limit of Tentative Dietary Goal for Preventing Lifestyle-related Diseases; MUFA, monounsaturated fatty acids; PUFA, polyunsaturated fatty acids; RDA, Recommended Dietary Allowance; SFA, saturated fatty acids; ULDG, upper limit of Tentative Dietary Goal for Preventing Lifestyle-related Diseases; y, years. [1] Cutoff values were determined by the World Health Organization's definition [36]. [2] Cutoff values were determined based on dietary intake data obtained from 639 Japanese adults aged 20-81 y [25,28]. [3] Cutoff values were based on the scoring system of Healthy Eating Index-2015 [8]: <50%, ≥50% to <90%, and ≥90% of the maximum score for "improvement strongly recommended", "improvement recommended", and "good; no change recommended", respectively. For dairy, the cutoff values on the HEI-2015 system were halved given the large difference in the reference values for calcium between Japan and the US (Estimated Average Requirement: 500-650 vs 800-1100 mg/day; Recommended Dietary Allowance: 600-800 vs 1000-1300 mg/day) [10,37]. [4] Cutoff values were based on dietary reference values (see Table S3) in the Japanese Dietary Reference Intakes [10], unless otherwise indicated. [5] Cutoff values were determined by the World Health Organization's definition [38]. [6] Cutoff values were based a combined analysis of 83 prospective cohort studies on the association between alcohol consumption and mortality [39].

Table S3. Dietary reference values used in the personalized nutrition system [1].

	**18-29 y**				**30-49 y**				**50-64 y**				**65-74 y**				**≥75 y**			
	**EAR**	**RDA**	**AI**	**DG**	**EAR**	**RDA**	**AI**	**DG**	**EAR**	**RDA**	**AI**	**DG**	**EAR**	**RDA**	**AI**	**DG**	**EAR**	**RDA**	**AI**	**DG**
Men																				
Protein (g/d)	50	65	---	---	50	65	---	---	50	65	---	---	50	60	---	---	50	60	---	---
Protein (% of total energy)	---	---	---	13-20	---	---	---	13-20	---	---	---	14-20	---	---	---	15-20	---	---	---	15-20
Fat (% of total energy)	---	---	---	20-30	---	---	---	20-30	---	---	---	20-30	---	---	---	20-30	---	---	---	20-30
SFA (% of total energy)	---	---	---	≤7	---	---	---	≤7	---	---	---	≤7	---	---	---	≤7	---	---	---	≤7
n-3 PUFA (g/d)	---	---	2	---	---	---	2	---	---	---	2.2	---	---	---	2.2	---	---	---	2.1	---
n-6 PUFA (g/d)	---	---	11	---	---	---	10	---	---	---	10	---	---	---	9	---	---	---	8	---
Carbohydrate (% of total energy)	---	---	---	50-65	---	---	---	50-65	---	---	---	50-65	---	---	---	50-65	---	---	---	50-65
Dietary fiber (g/d)	---	---	---	≥21	---	---	---	≥21	---	---	---	≥21	---	---	---	≥20	---	---	---	≥20
Sodium (NaCI g/d)	---	---	---	<7.5	---	---	---	<7.5	---	---	---	<7.5	---	---	---	<7.5	---	---	---	<7.5
Potassium (mg/d)	---	---	---	≥3000	---	---	---	≥3000	---	---	---	≥3000	---	---	---	≥3000	---	---	---	≥3000
Calcium (mg/d)	650	800	---	---	600	750	---	---	600	750	---	---	600	750	---	---	600	700	---	---
Magnesium (mg/d)	280	340	---	---	310	370	---	---	310	370	---	---	290	350	---	---	270	320	---	---
Phosphorus (mg/d)	---	---	1000	---	---	---	1000	---	---	---	1000	---	---	---	1000	---	---	---	1000	---
Iron (mg/d)	6.5	7.5	---	---	6.5	7.5	---	---	6.5	7.5	---	---	6	7.5	---	---	6	7	---	---
Zinc (mg/d)	9	11	---	---	9	11	---	---	9	11	---	---	9	11	---	---	9	10	---	---
Copper (mg/d)	0.7	0.9	---	---	0.7	0.9	---	---	0.7	0.9	---	---	0.7	0.9	---	---	0.7	0.8	---	---
Manganese (mg/d)	---	---	4	---	---	---	4	---	---	---	4	---	---	---	4	---	---	---	4	---
Vitamin A (μg/d)	600	850	---	---	650	900	---	---	650	900	---	---	600	850	---	---	550	800	---	---
Vitamin D (μg/d)	---	---	8.5	---	---	---	8.5	---	---	---	8.5	---	---	---	8.5	---	---	---	8.5	---
Vitamin E (mg/d)	---	---	6	---	---	---	6	---	---	---	7	---	---	---	7	---	---	---	6.5	---
Vitamin K (μg/d)	---	---	150	---	---	---	150	---	---	---	150	---	---	---	150	---	---	---	150	---
Thiamin (mg/d)	1.2	1.4	---	---	1.2	1.4	---	---	1.1	1.3	---	---	1.1	1.3	---	---	1	1.2	---	---
Riboflavin (mg/d)	1.3	1.6	---	---	1.3	1.6	---	---	1.2	1.5	---	---	1.2	1.5	---	---	1.1	1.3	---	---
Niacin (mg/d)	13	15	---	---	13	15	---	---	12	14	---	---	12	14	---	---	11	13	---	---
Vitamin B-6 (mg/d)	1.1	1.4	---	---	1.1	1.4	---	---	1.1	1.4	---	---	1.1	1.4	---	---	1.1	1.4	---	---
Vitamin B-12 (μg/d)	2	2.4	---	---	2	2.4	---	---	2	2.4	---	---	2	2.4	---	---	2	2.4	---	---
Folate (μg/d)	200	240	---	---	200	240	---	---	200	240	---	---	200	240	---	---	200	240	---	---
Pantothenic acid (mg/d)	---	---	5	---	---	---	5	---	---	---	6	---	---	---	6	---	---	---	6	---

Vitamin C (mg/d)	85	100	---	---	85	100	---	---	85	100	---	---	80	100	---	---	80	100	---	---
Women																				
Protein (g/d)	40	50	---	---	40	50	---		40	50	---	---	40	50	---	---	40	50	---	---
Protein (% of total energy)	---	---	---	13-20	---	---	---	13-20	---	---	---	14-20	---	---	---	15-20	---	---	---	15-20
Fat (% of total energy)	---	---	---	20-30	---	---	---	20-30	---	---	---	20-30	---	---	---	20-30	---	---	---	20-30
SFA (% of total energy)	---	---	---	≤7	---	---	---	≤7	---	---	---	≤7	---	---	---	≤7	---	---	---	≤7
n-3 PUFA (g/d)	---	---	1.6	---	---	---	1.6	---	---	---	1.9	---	---	---	2	---	---	---	1.8	---
n-6 PUFA (g/d)	---	---	8	---	---	---	8	---	---	---	8	---	---	---	9	---	---	---	8	---
Carbohydrate (% of total energy)	---	---	---	50-65	---	---	---	50-65	---	---	---	50-65	---	---	---	50-65	---	---	---	50-65
Dietary fiber (g/d)	---	---	---	≥18	---	---	---	≥18	---	---	---	≥18	---	---	---	≥17	---	---	---	≥17
Sodium (NaCl g/d)	---	---	---	<6.5	---	---	---	<6.5	---	---	---	<6.5	---	---	---	<6.5	---	---	---	<6.5
Potassium (mg/d)	---	---	---	≥2600	---	---	---	≥2600	---	---	---	≥2600	---	---	---	≥2600	---	---	---	≥2600
Calcium (mg/d)	550	650	---	---	550	650	---	---	550	650	---	---	550	650	---	---	500	600	---	---
Magnesium (mg/d)	230	270	---	---	240	290	---	---	240	290	---	---	230	280	---	---	220	260	---	---
Phosphorus (mg/d)	---	---	800	---	---	---	800	---	---	---	800	---	---	---	800	---	---	---	800	---
Iron (mg/d)	8.5	10.5	---	---	9	10.5	---	---	9	11	---	---	5	6	---	---	5	6	---	---
Zinc (mg/d)	7	8	---	---	7	8	---	---	7	8	---	---	7	8	---	---	6	8	---	---
Copper (mg/d)	0.6	0.7	---	---	0.6	0.7	---	---	0.6	0.7	---	---	0.6	0.7	---	---	0.6	0.7	---	---
Manganese (mg/d)	---	---	3.5	---	---	---	3.5	---	---	---	3.5	---	---	---	3.5	---	---	---	3.5	---
Vitamin A (μg/d)	450	650	---	---	500	700	---	---	500	700	---	---	500	700	---	---	450	650	---	---
Vitamin D (μg/d)	---	---	8.5	---	---	---	8.5	---	---	---	8.5	---	---	---	8.5	---	---	---	8.5	---
Vitamin E (mg/d)	---	---	5	---	---	---	5.5	---	---	---	6	---	---	---	6.5	---	---	---	6.5	---
Vitamin K (μg/d)	---	---	150	---	---	---	150	---	---	---	150	---	---	---	150	---	---	---	150	---
Thiamin (mg/d)	0.9	1.1	---	---	0.9	1.1	---	---	0.9	1.1	---	---	0.9	1.1	---	---	0.8	0.9	---	---
Riboflavin (mg/d)	1	1.2	---	---	1	1.2	---	---	1	1.2	---	---	1	1.2	---	---	0.9	1	---	---
Niacin (mg/d)	9	11	---	---	10	12	---	---	9	11	---	---	9	11	---	---	9	10	---	---
Vitamin B-6 (mg/d)	1	1.1	---	---	1	1.1	---	---	1	1.1	---	---	1	1.1	---	---	1	1.1	---	---
Vitamin B-12 (μg/d)	2	2.4	---	---	2	2.4	---	---	2	2.4	---	---	2	2.4	---	---	2	2.4	---	---
Folate (μg/d)	200	240	---	---	200	240	---	---	200	240	---	---	200	240	---	---	200	240	---	---
Pantothenic acid (mg/d)	---	---	5	---	---	---	5	---	---	---	5	---	---	---	5	---	---	---	5	---
Vitamin C (mg/d)	85	100	---	---	85	100	---	---	85	100	---	---	80	100	---	---	80	100	---	---

AI, Adequate Intake; d, day; DG, Tentative Dietary Goal for Preventing Lifestyle-related Diseases; EAR, Estimated Average Requirement; PUFA, polyunsaturated fatty acids; RDA, Recommended Dietary Allowance; SFA, saturated fatty acids; y, years. [1] All values are derived from the Japanese Dietary Reference Intakes [10].

Here is a detailed summary of your diet.

Date of data analysis: 19/09/2021

We used numbers to show how you eat and compared them to the reference values. The reference value is the amount of food you should eat to prevent nutritional deficiencies and lifestyle-related diseases, based on your sex and age. The items marked with ○ are those that meet the reference values, so let's continue with the current way of eating. Items marked with △ are those that almost meet the reference, and those marked with × are those that are far from the reference. Let's start improving where we can.

Section 4 also shows major food sources of each of nutrients in Japanese. If you find that you are getting too much of a nutrient, you should reduce the frequency of eating the source food or the amount you eat at one time. For a nutrient judged to be low, it is recommended to increase the frequency of eating the source foods and the amount eaten at one time.

ID	****
Sex	Male
Date of birth	****
Date of data entry	29/01/2021
Age (years)	35
Body height (cm)	166.0
Body weight (kg)	57.0
BMI (kg/m^2)	20.7
Pregnancy/lactation	---
Estimated energy requirement (kcal/d)	2700
Repoted energy intake (kcal/d)	1845
Energy intake: energy requirement	0.68
Dietary reporting status*	Underreport

* Note: The amount of food calculated from your answers was much less than the amount of food that would maintain your weight. Although we have compensated for this, the results shown here may differ from the way you actually eat, so please consider them as "approximate results" only.

Section 1: body weight

It is important to exercise as well as eat to manage your weight.

	Your BMI	Your grade	Explanation
BMI (kg/m^2)	20.7 (Ref: 18.5-25)	○ Normal weight	BMI is calculated as your weight (kg) divided by your height (m) squared, which indicates how healthy your weight is for your height. Measure and monitor your weight regularly in your daily life.

BMI: body mass index. Assessed based on the WHO definition.

Section 2: eating patterns

Improve the quality of your diet by reviewing how you eat foods and nutrients.

	Your diet	Your grade	Explanation
Meal freqency (times/d)	0.0 (Ref: 3)	× Too little	It has been scientifically proven that eating three meals (breakfast, lunch, and dinner) properly is associated with favorable nutrient intake patterns. Try not to skip meals whenever possible.
Energy from breakfast (%)	19.6 (Ref: ≥20)	△ A little less	The optimal distribution of energy (calories) among the three meals (breakfast, lunch, and dinner) has not yet been clarified. The graph shows the average energy allocation for people of the same gender and age as you. It is clear that eating three proper meals is associated with favorable nutrient intake patterns, and conversely, skipping meals (especially breakfast) is associated with unfavorable nutrient intake patterns. Not skipping meals is more important than the distribution of each.
Energy from lunch (%)	16.5 (Ref: 25-40)	△ A little less	
Energy from dinner (%)	42.3 (Ref: 30-45)	○ Just right	
Energy from snacks (%)	21.6 (Ref: <10)	× Too much	Snacking is not bad itself, but too much snacking can have a negative impact on the three meals (breakfast, lunch, and dinner). Just be careful not to eat or drink too much of anything that contains too much energy, and enjoy your snacks in moderation!
Quality of breakfast (Max: 100)	57.3 (Ref: ≥70)	△ A little low	The "Healthy Eating Index-2015" is used to evaluate the quality of the diet. This is a 100-point scale that assesses the overall quality of the 10 most important foods and nutrients in the diet. The higher the score, the lower the risk of lifestyle-related diseases such as diabetes and heart disease. The 10 items are fruits, vegetables, dairy products, protein sources, whole grains, refined grains, sodium, saturated fatty acids, ratio of unsaturated to saturated fatty acids, and added sugars. For the average Japanese, the nutritional quality is highest for dinner, followed by lunch, breakfast, and snacks (see the graph). Here, the reference value was set at 70 points. This is a bit strict, but how was the quality of your meals? To improve the quality of your diet, let's check the above 10 items in Sections 3 and 4, and work on improving them one at a time, starting with the ones you think you can do.
Quality of lunch (Max: 100)	34.2 (Ref: ≥70)	× Low	
Quality of dinner (Max: 100)	62.5 (Ref: ≥70)	△ A little low	
Quality of snacks (Max: 100)	13.7 (Ref: ≥70)	× Low	
Quality of overall diet (Max: 100)	57.5 (Ref: ≥70)	△ A little low	

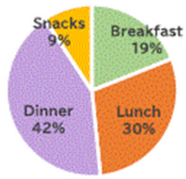

Average for Japanese of the same age and gender as you (men in their 40s)

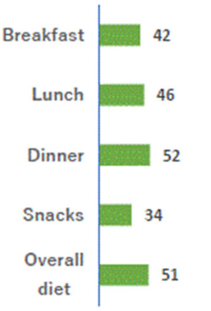

Average for Japanese of the same age and gender as you (men in their 40s)

Reference values based on diet reported by 639 healthy Japanese aged 20-81 y. (MDHQ feedback report 2/6)

© 2021 Kentaro Murakami

Figure S1. Part 2 (pages 2-4) of the dietary feedback report. This is a quantitative assessment of all dietary components. There are four sections in this part: Section 1: weight status; Section 2: eating patterns; Section 3: food group intake; Section 4: nutrient intake. All the variables assessed here as well as the assessment criteria are shown in Table S2. Here, only the second page of the translated version is shown; the original is in Japanese.

For those who want to know more

This is a summary of your food consumption.

Use this as a resource to help you figure out where you are getting too much of the foods to decrease, and where you can increase the amount of foods to increase.

MDHQ
Meal-based Diet History Questionnaire

* Note: The amount of food calculated from your answers was much less than the amount of food that would maintain your weight. Although we have compensated for this, the results shown here may differ from the way you actually eat, so please consider them as "approximate results" only.

Date of data analysis: 19/09/2021

ID	****
Sex	Male
Date of birth	****
Date of data entry	29/01/2021
Age (years)	42
Body height (cm)	166.0
Body weight (kg)	57.0
BMI (kg/m^2)	20.7
Pregnancy/lactation	---
Estimated energy requirement (kcal/d)	2700
Repoted energy intake (kcal/d)	1845
Energy intake: energy requirement	0.68
Dietary reporting status *	Underreport

Daily eating frequency	
Breakfast	0.0
Morning snack	0.4
Lunch	0.0
Afternoon snack	1.0
Dinner	0.0
Night snack	0.6
Meals	0.0
Snacks	2.0
Total	2.0

Behavior	Answer
Fatty meat	Sometimes
Taste strength	A little weak
Soy sauce (frequency)	Rarely
Soy sauce (amount)	very small
Amont of dishes	same as eating out
Amount of rice	relatively large at home

Meat	Answer
Grill	1/wk
Stew	2-3/wk
Fry	<1/wk
Stir fry	4-6/wk
Wafu	2-3/wk

Fish	Answer
Raw	1/wk
Grill	2-3/wk
Stew	1/wk
Fry	1/wk

		Breakfast		Morning snack		Lunch		Afternoon snack		Dinner		Night snack		Total
Food	Answer	Answer	g/d	Answer	g/d	Answer	g/d	Answer	g/d	Answer	g/d	Answer	g/d	g/d
Rice		2/wk	71	---	0	1/wk	45	---	0	4/wk	164	---	0	279
White rice	---	---	65	---	0	---	41	---	0	---	151	---	0	257
Brown rice	Sometimes/20%	---	6	---	0	---	4	---	0	---	13	---	0	22
Bread	Wholemeal bread: rarely	4/wk	69	---	0	1/wk	20	---	0	2/wk	29	---	0	118
Noodles		0/wk	0	---	0	3/wk	116	---	0	1/wk	36	---	0	153
Wheat noodles	Rarely	---	0	---	0	---	8	---	0	---	3	---	0	10
Chinese noodles	Sometimes	---	0	---	0	---	46	---	0	---	14	---	0	60
Instant noodles	Rarely	---	0	---	0	---	1	---	0	---	0	---	0	2
Spaghetti	Often	---	0	---	0	---	59	---	0	---	20	---	0	78
Buckwheat noodles	Rarely	---	0	---	0	---	2	---	0	---	1	---	0	3
Breakfast cereals		1/wk	13	---	0	---	0	---	0	---	0	---	0	13
Miso soup		0/wk	0	---	0	0/wk	0	---	0	1/wk	38	---	0	38
Pickled vegetables		0/wk	0	---	0	0/wk	0	---	0	1/wk	5	---	0	5
Meat		0/wk	0	---	0	3/wk	31	---	0	6/wk	96	---	0	127
Liver	Never	---	0	---	0	---	0	---	0	---	0	---	0	0
Processed meat	Sometimes	---	0	---	0	---	5	---	0	---	6	---	0	11
Beef	Rarely	---	0	---	0	---	0	---	0	---	2	---	0	3
Chicken	Often	---	0	---	0	---	17	---	0	---	61	---	0	78
Pork	Sometimes	---	0	---	0	---	8	---	0	---	27	---	0	36
Fish and shellfish		0/wk	0	---	0	0/wk	0	---	0	5/wk	94	---	0	94
Small fish with bones	Rarely	---	0	---	0	---	0	---	0	---	0	---	0	0
Dried fish	Never	---	0	---	0	---	0	---	0	---	0	---	0	0
Canned tuna	Rarely	---	0	---	0	---	0	---	0	---	0	---	0	0
Salmon	Sometimes	---	0	---	0	---	0	---	0	---	11	---	0	11
White meat fish	Sometimes	---	0	---	0	---	0	---	0	---	25	---	0	25
Oily fish	Sometimes	---	0	---	0	---	0	---	0	---	25	---	0	25
Red meat fish	Sometimes	---	0	---	0	---	0	---	0	---	21	---	0	21
Squid and octopus	Rarely	---	0	---	0	---	0	---	0	---	1	---	0	1
Eel	Rarely	---	0	---	0	---	0	---	0	---	0	---	0	0
Shrimp and crab	Rarely	---	0	---	0	---	0	---	0	---	1	---	0	1
Shellfish	Sometimes	---	0	---	0	---	0	---	0	---	8	---	0	8
Fish eggs	Never	---	0	---	0	---	0	---	0	---	0	---	0	0
Ground fish meat products	Rarely	---	0	---	0	---	0	---	0	---	1	---	0	1
Eggs		0/wk	0	---	0	0/wk	0	---	0	1/wk	6	---	0	6
Dairy products		5/wk	143	0/wk	0	0/wk	0	0/wk	0	1/wk	16	0/wk	0	159
Low-fat milk	Rarely	---	1	---	0	---	0	---	0	---	0	---	0	1
Full-fat milk	Often	---	80	---	0	---	0	---	0	---	9	---	0	89
Yogurt	Sometimes	---	61	---	0	---	0	---	0	---	6	---	0	67
Cheese	Rarely	---	0	---	0	---	0	---	0	---	0	---	0	0
Ice cream	Sometimes	---	0	---	0	---	0	---	0	---	1	---	0	1
Pulses and nuts		1/wk	9	---	0	0/wk	0	---	0	3/wk	40	---	0	50
Tofu (i.e., soybean curd)	Sometimes	---	5	---	0	---	0	---	0	---	37	---	0	42
Natto (i.e., fermented soybeans)	Sometimes	---	3	---	0	---	0	---	0	---	2	---	0	6
Tofu products	Rarely	---	0	---	0	---	0	---	0	---	1	---	0	1
Soy milk	Never	---	0	---	0	---	0	---	0	---	0	---	0	0
Nuts (including peanuts)	Sometimes	---	0	---	0	---	0	---	0	---	1	---	0	1

(Continues on next page)
(MDHQ feedback report 5/6)
© 2021 Kentaro Murakami

Figure S2. Part 3 (pages 5-6) of the dietary feedback report. This is a quantitative information on food intake for each eating occasion. This part includes information on dietary intake of 24 generic food groups and 88 food items (see Table S1). All intake values are shown for breakfast, morning snack, lunch, afternoon snack, dinner, and night snack separately, as well as for the total amount from all eating occasions. Here, only the fifth page of the translated version is shown; the original is in Japanese.

Table S4. Behavior change techniques (BCTs) used in the personalized nutrition system.

BCT code [1]				BCT label	Selected/not selected	Dietary feedback report	Other aspects (beyond the scope of this study) [2]
MDHQ	Food4Me	CALO-RE	SC				
1	1	1	---	Provide information on consequences of behavior in general	Selected	Part 2: descriptions on foods and nutrients	---
2	2	2	---	Provide information on consequences of behavior to the individual	Selected	Part 1: descriptions on sodium, saturated fats, and added sugars	---
3	3	3	---	Provide information about others' approval	Selected	Part 1: personalized messages such as "You are doing really well!"	---
4	4	13	---	Provide rewards contingent on successful behavior	Selected	Part 1: personalized messages such as "Keep it as it is."	---
---	5	14	---	Shaping	Not selected	---	---
5	6	19	---	Provide feedback on performance	Selected	Parts 1-3: feedback report itself	---
6	7	5	---	Goal setting (behavior)	Selected	Part 1: indicating "Big 3" (three most relevant components); Part 2: reference values of dietary intakes	---
7	8	6	---	Goal setting (outcome)	Selected	Part 2: reference value of weight status	---
8	9	9	---	Set graded tasks	Selected	Part 1: personalized messages such as "Try to have as much amount of soups left as you can."	---
9	10	10	---	Prompt review of behavioral goals	Selected	Parts 1 and 2: assessment (signal) for dietary variables	---
10	11	11	---	Prompt review of outcome goals	Selected	Parts 1 and 2: assessment (signal) for weight status	---
11	12	17	---	Prompt self-monitoring of behavioral outcome	Selected	Part 2: message on weight status (i.e., "Take regular weight measurements.")	---
12	13	7	---	Action planning	Selected	Part 1: personalized messages on major sources of sodium, saturated fats, and added sugars such as "Your major food source of added sugars is confectioneries. As some of the confectioneries are high in added sugars, eat them in moderation and take care with your portion size. Swap confectioneries for fruit, low-fat dairy products, and nuts."	---
13	14	8	---	Barrier identification or problem solving	Selected	Part 1: personalized messages on major sources of sodium, saturated fats, and added sugars such as "Your major food source of saturated fat is meat. Trim the fat of meat before cooking or eating it. Reduce your intake of processed meat such as sausage and bacon. Swap meat for fish."	---
14	15	26	---	Prompt practice	Selected	Part 1: personalized messages on major sources of sodium, saturated fats, and added sugars such as "Try to use smaller amounts of salt-	---

						based seasonings. Swap salt-based seasonings for lemon juice, vinegar, spices, and herbs. Select low-salt seasonings whenever possible.”	
---	16	29	---	Plan social support or social change	Not selected	---	---
15	17	---	RD2	Emphasize choice	Selected	Part 1: a general message “Let’s work on each one from where it seems possible.”	---
16	18	---	RD1	Tailor interactions appropriately	Selected	Parts 1-3: feedback report created based on dietary intake data	---
17	19	---	RI1	Assess current and past dietary behavior	Selected	Not used	When repeated feedbacks are possible, the second feedback report will include dietary feedback in comparison with the first report
18	20	---	RI2	Assess current readiness and ability to change	Selected	Not used	Maybe assessed at baseline or screening
19	21	---	RI3	Assess past history of dietary change attempts	Selected	Not used	Could be done in repeated design
20	22	---	RI4	Assess adverse reactions	Selected	Not used	Will be monitored throughout a trial
21	23	21	---	Provide instruction on how to perform the behavior	Selected	Parts 1-2: provide tips on how to achieve dietary targets	---
22	24	40	---	Stimulate anticipation of future rewards	Selected	Not used	Encouraging continued participation and data collection; promising provision of dietary feedback
---	25	27	---	Use of follow-up prompts	Not selected	---	---
23	26	20	---	Provide information on where and when to perform the behavior	Selected	Part 1: personalized messages such as “Fruit can be included in your snack list and eaten after meals.”	---
24	---	4	---	Provide formative information about others’ behavior	Selected	Part 2: showing the mean contribution of each meal to total energy and the mean diet quality score of general adults with same age and sex of the participant	---

CALO-RE, Coventry, Aberdeen, and London-Refined; RD, general aspects of the interaction (R) focusing on delivery of the intervention (D); RI, general aspects of the interaction (R) focusing on information gathering (I); SC, smoking cessation. [1] These are BCT codes used in each of the previous studies [11,17,18], as well as in the present study (MDHQ columun), which are just shown for future references. [2] Will be considered for a future intervention trial.

Table S5. Acceptability scores of the MDHQ feedback report according to dietary reporting status, whether the feedback report was printed, and time spent reading the feedback report [1].

Variable	Dietary reporting status		Whether the MDHQ feedback report was printed		Time spent reading the MDHQ feedback report	
	Implausible [2] (n = 65)	Plausible [3] (n = 190)	No (n = 133)	Yes (n = 122)	Less than 20 min (n = 162)	20 min or more (n = 93)
Total score	4.1 ± 0.4	4.3 ± 0.4 **	4.2 ± 0.4	4.3 ± 0.4 **	4.2 ± 0.4	4.3 ± 0.4 *
The report is believable	4.0 ± 0.6	4.2 ± 0.7	4.1 ± 0.6	4.2 ± 0.6	4.1 ± 0.7	4.2 ± 0.5 *
The report is relevant	4.1 ± 0.7	4.4 ± 0.6 **	4.2 ± 0.7	4.4 ± 0.6 *	4.2 ± 0.7	4.4 ± 0.6 *
The report is interesting	4.4 ± 0.6	4.7 ± 0.5 **	4.5 ± 0.6	4.7 ± 0.5 *	4.5 ± 0.6	4.7 ± 0.5
The report is logical	4.1 ± 0.7	4.3 ± 0.6 **	4.2 ± 0.6	4.4 ± 0.6 *	4.2 ± 0.6	4.4 ± 0.7
The report is understandable	4.3 ± 0.7	4.3 ± 0.6	4.3 ± 0.7	4.3 ± 0.6	4.3 ± 0.7	4.3 ± 0.6
The report is well formulated	4.3 ± 0.7	4.5 ± 0.7	4.4 ± 0.7	4.4 ± 0.7	4.4 ± 0.7	4.5 ± 0.7
The report is complete	4.3 ± 0.7	4.5 ± 0.6 **	4.4 ± 0.7	4.5 ± 0.6	4.4 ± 0.7	4.6 ± 0.6 *
The report is too long	3.2 ± 1.0	3.3 ± 1.1	3.2 ± 1.0	3.3 ± 1.1	3.3 ± 1.0	3.2 ± 1.1
The report is personal	4.3 ± 0.8	4.5 ± 0.7 *	4.3 ± 0.8	4.5 ± 0.6 *	4.4 ± 0.8	4.5 ± 0.6
The report is correct	3.7 ± 0.8	4.0 ± 0.7 **	3.8 ± 0.7	4.0 ± 0.7 *	3.8 ± 0.7	4.0 ± 0.7 *
I will use the report	4.2 ± 0.8	4.3 ± 0.7	4.2 ± 0.7	4.4 ± 0.7 *	4.2 ± 0.7	4.5 ± 0.6 **
The report will help me to eat healthier	4.5 ± 0.6	4.5 ± 0.6	4.4 ± 0.6	4.6 ± 0.6 *	4.5 ± 0.6	4.6 ± 0.5

MDHQ, Meal-based Diet History Questionnaire. [1] Values are means ± standard deviations. Acceptability scores range from 1 ("strongly disagree") to 5 ("strongly agree"), except for the item "The report is too long" for which the score is assigned vice versa. The total score was calculated as the average of all 12 items. Mean values between the two categories of each variable were compared using independent t-test: * $p < 0.05$; ** $p < 0.01$. [2] Including 63 under-reporters and 2 over-reporters. Under- and over-reporters were defined as participants with the ratio of reported energy intake to estimated energy requirement of <0.7 and >1.3, respectively. [3] Defined as participants with the ratio of reported energy intake to estimated energy requirement of 0.7 to 1.3.

Table S6. Acceptability scores of the MDHQ feedback report according to age, weight status, and diet quality [1].

Variable	Age [2]		Weight status [3]			Diet quality [4]	
	Younger (n = 129)	Older (n = 126)	Underweight (n = 27)	Normal weight (n = 183)	Overweight (n = 45)	Lower (n = 128)	Higher (n = 127)
Total score	4.2 ± 0.4	4.3 ± 0.4	4.2 ± 0.5	4.3 ± 0.4	4.2 ± 0.5	4.2 ± 0.4	4.3 ± 0.4
The report is believable	4.1 ± 0.6	4.2 ± 0.6	4.1 ± 0.7	4.1 ± 0.6	4.1 ± 0.6	4.1 ± 0.6	4.1 ± 0.6
The report is relevant	4.2 ± 0.7	4.4 ± 0.6	4.3 ± 0.7	4.3 ± 0.6	4.2 ± 0.7	4.3 ± 0.7	4.3 ± 0.6
The report is interesting	4.6 ± 0.6	4.6 ± 0.5	4.6 ± 0.6	4.6 ± 0.5	4.6 ± 0.5	4.5 ± 0.6	4.6 ± 0.5
The report is logical	4.2 ± 0.6	4.4 ± 0.7	4.3 ± 0.6	4.3 ± 0.7	4.2 ± 0.6	4.2 ± 0.6	4.3 ± 0.7
The report is understandable	4.2 ± 0.7	4.4 ± 0.6 **	3.9 ± 0.7 [a]	4.4 ± 0.6 [b]	4.2 ± 0.7 [ab]	4.3 ± 0.6	4.3 ± 0.7
The report is well formulated	4.4 ± 0.7	4.4 ± 0.7	4.1 ± 0.8 [a]	4.4 ± 0.7 [b]	4.4 ± 0.6 [ab]	4.4 ± 0.7	4.4 ± 0.7
The report is complete	4.5 ± 0.7	4.5 ± 0.6	4.4 ± 0.6	4.5 ± 0.6	4.4 ± 0.7	4.5 ± 0.6	4.5 ± 0.7
The report is too long	3.1 ± 1.1	3.4 ± 1.0 *	2.8 ± 1.1 [a]	3.3 ± 1.0 [ab]	3.5 ± 1.2 [b]	3.2 ± 1.0	3.3 ± 1.1
The report is personal	4.4 ± 0.7	4.4 ± 0.7	4.4 ± 0.7	4.5 ± 0.7	4.3 ± 0.9	4.4 ± 0.8	4.5 ± 0.7
The report is correct	3.8 ± 0.7	4.0 ± 0.7	4.0 ± 0.7	3.9 ± 0.7	4.0 ± 0.7	3.9 ± 0.7	4.0 ± 0.7
I will use the report	4.3 ± 0.7	4.3 ± 0.7	4.3 ± 0.8	4.3 ± 0.6	4.4 ± 0.8	4.3 ± 0.7	4.4 ± 0.7
The report will help me to eat healthier	4.5 ± 0.6	4.5 ± 0.6	4.6 ± 0.6 [ab]	4.6 ± 0.5 [a]	4.3 ± 0.7 [b]	4.5 ± 0.6	4.6 ± 0.6

MDHQ, Meal-based Diet History Questionnaire. [1] Values are means ± standard deviations. Acceptability scores range from 1 ("strongly disagree") to 5 ("strongly agree"), except for the item "The report is too long" for which the score is assigned vice versa. The total score was calculated as the average of all 12 items. Mean values between the two categories of age and diet quality were compared using independent *t*-test: * $p < 0.05$; ** $p < 0.01$. For weight status, mean values between the three categories were compared using analysis of variance. When the overall p from analysis of variance was <0.05, Bonferroni's post hoc test was performed; values within each variable with unlike superscript letters are significantly different ($p < 0.05$). [2] Two categories were created by median age (48 years). [3] Weight status was categorized on the basis of body mass index (in kg/m^2): underweight (<18.5), normal weight (≥18.5 to <25), or overweight (≥25). [4] Diet quality was assessed by the Healthy Eating Index-2015 total score; two categories were created by median score (56.8).
